# Mobilization of a Simulation Platform to Facilitate a System-wide Response to the COVID-19 Pandemic

**DOI:** 10.5811/westjem.2020.6.47837

**Published:** 2020-06-23

**Authors:** David J. Carlberg, Tiffany M. Chan, Diana Ladkany, Jessica Palmer, Kevin Bradshaw

**Affiliations:** * Georgetown University School of Medicine, MedStar Georgetown University Hospital, Department of Emergency Medicine, Washington, District of Columbia; † MedStar Harbor Hospital, Department of Emergency Medicine, Baltimore, Maryland; ‡ Georgetown University School of Medicine, MedStar Washington Hospital Center, Department of Emergency Medicine, Washington, District of Columbia; § Georgetown University School of Medicine, MedStar Southern Maryland Hospital Center, Department of Emergency Medicine, Prince George’s County, Maryland; ¶ MedStar Health Simulation Training and Education Lab, Washington, District of Columbia

## INTRODUCTION

While simulation plays a prominent role in healthcare education at every level,^1^ the ability to perform traditional, in-person simulation has been practically eliminated by the severe acute respiratory syndrome coronavirus 2 (SARS-CoV), or COVID-19, pandemic. Simultaneously, COVID-19-related education has become vital, as providers work to expand their knowledge base and learn new skills. Were it not for social distancing, simulation would play a major role in addressing the pandemic’s challenges. Simulation-based education could help providers optimize patient care while minimizing viral aerosolization. Simulation could even teach strategies for coping with the emotional consequences of working during a pandemic.

Despite limitations on traditional operations, simulation platforms should explore opportunities to support the COVID-19 pandemic response. On a basic level, active engagement in the response helps keep simulation programs relevant. On a more idealistic level, simulation can play an important role in combating one of the greatest human challenges in recent memory. With modifications, versions of traditional simulation education can continue: some activities can move to videoconference, and mannequins can be distributed to clinical areas for in situ education. The pandemic also provides opportunities for simulation services to move beyond classic roles: simulation labs have medical supplies that can be repurposed clinically, and simulation specialists have unique skill sets that can be applied outside the simulation lab.

In many ways, the simulation community has already shown its utility during the pandemic response, highlighting the utility of simulation for optimizing personal protective equipment (PPE) utilization and evaluating the readiness of a COVID-19 inpatient unit.^2^,^3^,^4^ We discuss strategies our simulation platform used to work synergistically with our emergency departments (ED) and our health system to address the COVID-19 pandemic. (Table 1) We hope this discussion stimulates ideas for unique and unexpected ways resources can be leveraged as part of the pandemic response.

## STRATEGIES

### Development of COVID-19-specific Simulation Modules for Emergency Medicine Residents

The value of simulation to emergency medicine (EM) residents during the pandemic is multifold: it allows them to develop and hone new skills for managing COVID-19 patients without risking viral exposure and compromising personal safety. We developed a simulation scenario featuring a COVID-19 patient with progressive respiratory distress. This low-fidelity simulation required only a videoconferencing platform, a rudimentary monitor, and the simulation moderator.

Residents were divided into groups of ~5 learners, and each group worked through the case, explaining COVID-19-specific interventions step-by-step. They had to identify the patient’s high risk for decompensation, escalate oxygen therapy, highlight opportunities for reducing virus aerosolization, employ methods for awake proning, effectively preoxygenate, intubate, and subsequently manage the ventilator. Initial management was primarily performed by first-year residents; the peri-intubation phase was managed primarily by second-year residents; and the post-intubation phase was managed primarily by third-year residents. A debrief followed.

While EM resident simulations often push learners outside their comfort zones and force them to make important medical decisions in critically ill patients with limited information, this simulation was designed to provide learners with the confidence to manage the intricacies of respiratory failure due to COVID-19. The remote-learning format worked well because the simulation avoided a procedural focus and instead focused on differentiating COVID-19 management from classic acute respiratory distress syndrome management. The low-fidelity, distance-learning simulation was well received by residents and faculty moderators.

### Facilitating Self-Directed in Situ Simulation

Frequently, simulation training on new equipment happens in groups, either in the simulation lab or in situ. It is usually moderated by an instructor who has a deep-rooted understanding of best practices and who can provide real-time, constructive feedback to learners. Because of the pandemic, training on new equipment has required modification of this approach.

Our health system provided each ED with a plexiglass box designed to reduce droplet spread of the COVID-19 virus by encapsulating a patient’s head during intubation.^5^,^6^,^7^ With the controversy surrounding the utility of such barrier enclosure devices^8^,^9^,^10^,^11^ and with the changes in intubation mechanics required for their use, our providers could choose whether or not to incorporate the box into their clinical practice. To help providers assess the practicality of these devices and to allow providers the opportunity to practice with them, we deployed intubating heads, laryngoscopes, and endotracheal tubes to our departments for EM faculty and residents to practice with individually.

We also demonstrated a simulated intubation via videoconference, discussing changes in practice required by the box and highlighting potential challenges with its use. The session was recorded and subsequently distributed to our group via YouTube. By combining in situ, self-directed simulation with a recorded videoconference, we re-imagined how to train providers on newly introduced clinical equipment.

### Repurposing Simulation Equipment for Clinical Use

Simulation labs frequently obtain durable medical equipment in two ways: 1) They purchase state-of-the-art equipment from vendors; or 2) they receive equipment donated from the clinical arena when it has been replaced or become outdated. Either way, this equipment has little or no difference from equipment used clinically. As such, in a crisis it can be repurposed for clinical use.

Because of the importance of video laryngoscope (VL)-based airway management during the pandemic,^12^,^13^ obtaining additional VL devices became a top priority for our EDs. Pre-COVID-19, most of our EDs had one or two VLs, and many of the VL blades undergo sterile processing before re-use. The prospect of multiple simultaneous COVID-19-related VL intubations led us to strategically distribute our simulation lab’s four VLs among our EDs. Each laryngoscope was evaluated by the destination hospital’s biomedical engineering department, and each received certification for clinical use. Some required minor maintenance, such as battery replacement. Other simulation materials that could be deployed clinically include anesthesia machines, which can be used as ventilators, and personal protective equipment (PPE).

### Instructional Video Development for Telemedicine Provider Training

Because of the pandemic, use of telehealth in our EDs has expanded greatly: the number of EDs using a telehealth triage provider has increased, and EM telehealth providers may now discharge well-appearing, low-risk ED patients suspected to have COVID-19. These telehealth encounters vary significantly from in-person encounters: establishing rapport is different, and the evaluation relies heavily on the patient’s history, vital signs, and overall appearance. On a technological level, providers need to navigate new electronic interfaces as they progress through the patient encounter.

Our simulation-focused emergency physicians addressed these challenges by creating video-recorded simulated patient encounters via screen-capture software. The videos showed telehealth providers the steps required to start and stop the visits, as well as the key components for each type of visit. The video of a simulated ED triage encounter highlighted the brevity of these visits, as patients are seen in-person later in their ED stay. The videos simulating candidates for ED discharge highlighted the depth required by these visits, as well as reasons patients should be sent into the main ED for further evaluation. A separate training video was created showing the steps required on the triage nurse’s side of the telehealth interface. These videos were incorporated into telehealth training on the same day they were created.

### Exploring Donation Possibilities from Partner Companies

Simulation labs often work with private companies to design and purchase materials that improve the fidelity of simulation. Some of these relationships, especially with design and three-dimensional (3D) printing companies, can be longstanding and mutually beneficial. One of our partner 3D printing companies offered to donate thousands of face masks for clinicians. Exploring these partnerships for potential donations could be highly beneficial to health systems.

### Simulation Staff Redeployment to Support a Telemedicine Platform

In mid-March, our health system’s Simulation Training and Education Lab (SiTEL) canceled all classic simulation training. This was done, in part, for social distancing. Simultaneously, there was a greater need to support the system’s rapidly growing telemedicine platform. As EM-based telehealth expanded, outpatient clinics added virtual appointments, and urgent care telemedicine visits increased nearly 50-fold.

In response, SiTEL redeployed 23 full-time simulation, training, and education staff, along with 65 other full-time associates, into telehealth support roles. Despite a lack of significant prior telehealth experience, the staff refocused their training and education expertise over the course of six days. They developed a telehealth support center that has since trained over 12,000 healthcare providers on the equipment necessary to participate in telemedicine. Support center staff conduct test calls, ensuring providers can navigate telemedicine interfaces, have quality audiovisual connections, and have professional-appearing workspaces.

## CONCLUSION

During normal operations, simulation serves as a vital tool that allows learners to translate textbook concepts into safe, exceptional bedside care. Both social distancing and preparations for a pandemic caused a near-complete shutdown of classic simulation operations. Using our robust simulation platform, we supported our health network’s preparation for and management of the current pandemic. We used virtual and in situ simulation to prepare for critically ill COVID-19 patients; we repurposed simulation supplies for clinical use; we prepared healthcare providers to perform virtual evaluation and management of patients; we engaged vendors to obtain PPE; and we shifted our simulation staff’s focus to telemedicine. We hope that many of these strategies can be adopted in other EDs, hospitals, and health systems. Additionally, we hope this discussion stimulates ideas for how existing resources can be re-imagined and leveraged in response to the COVID-19 pandemic.

## Figures and Tables

**Table 1:** Simulation strategies to combat the COVID-19 pandemic.

Videoconference-based simulation for emergency medicine residents
Self-directed *in situ* simulation augmented by video for training on new equipment
Repurposing simulation equipment for clinical use
Development of training videos for a rapidly-expanding telemedicine platform
Exploring personal protective equipment donations from partner companies
Redeploying simulation specialists to support telemedicine endeavors
